# Supplementary material for: Genomic language model mitigates chimera artifacts in nanopore direct RNA sequencing
Source: Nat Commun. 2026 Jan 19;17:1864. doi: 10.1038/s41467-026-68571-5 (PMC12923543; doi:10.1038/s41467-026-68571-5)
Supplement: Supplementary file 1 — Supplementary Information [file 41467_2026_68571_MOESM1_ESM.pdf]

# Supplementary Information

Yangyang Li[1†], Ting–You Wang[1†], Qingxiang Guo[1], Yanan Ren[1], Xiaotong Lu[1], Qi Cao[1,2], Rendong Yang[1,2*]

[1]Department of Urology, Northwestern University Feinberg School of Medicine, 303 E Superior St, Chicago, 60611, IL, USA.
[2]Robert H. Lurie Comprehensive Cancer Center, Northwestern University Feinberg School of Medicine, 675 N St Clair St, Chicago, 60611, IL, USA.

*Corresponding author(s). E-mail(s): rendong.yang@northwestern.edu;
Contributing authors: yangyang.li@northwestern.edu; tywang@northwestern.edu; qingxiang.guo@northwestern.edu; ynren1020@gmail.com; xiaotong.lu@northwestern.edu; qi.cao@northwestern.edu;
†These authors contributed equally to this work.

**Supplementary Table 1** Summary of Adapter Trimming Tools for analyzing direct RNA sequencing (dRNA-seq) data

| **Adapter trimming tool** | **dRNA-seq terminal adapter trimming** | **dRNA-seq internal adapter trimming** | **Trimming existing dRNA-seq datasets (post-basecalling)** |
|---|---|---|---|
| Porechop | × | × | × |
| Porechop_ABI | × | × | × |
| Pychopper | × | × | × |
| Dorado | ✓ | × | × |
| DeepChopper | ✓ | ✓ | ✓ |

✓ indicates the tool supports this functionality; × indicates the tool does not support this functionality.

**Supplementary Table 2** Read Length Statistics by Sample

| Sample | Reads (M) | Min (bp) | Max (bp) | Mean (bp) | Std Dev (bp) | Q1 (bp) | Q2 (bp) | Q3 (bp) | P90 (bp) | P95 (bp) | P99 (bp) | Reads ≥32kb | % ≥32kb |
|---|---|---|---|---|---|---|---|---|---|---|---|---|---|
| A549 | 1.70 | 5 | 16,246 | 907 | 805 | 383 | 700 | 1,223 | 1,904 | 2,440 | 3,829 | 0 | 0 |
| MCF7 | 3.04 | 5 | 28,802 | 715 | 623 | 316 | 546 | 911 | 1,475 | 1,863 | 3,052 | 0 | 0 |
| HCT116 | 4.70 | 5 | 21,656 | 889 | 795 | 374 | 669 | 1,193 | 1,871 | 2,431 | 3,793 | 0 | 0 |
| K562 | 3.06 | 2 | 58,395 | 683 | 555 | 319 | 556 | 892 | 1,393 | 1,736 | 2,619 | 2 | 0 |
| HepG2 | 1.80 | 2 | 46,077 | 1,148 | 974 | 497 | 864 | 1,544 | 2,317 | 3,025 | 4,665 | 1 | 0 |
| VCaP RNA002 | 9.18 | 5 | 77,474 | 994 | 901 | 462 | 697 | 1,279 | 2,092 | 2,826 | 4,399 | 1 | 0 |
| VCaP RNA004 | 11.72 | 5 | 225,798 | 995 | 971 | 483 | 695 | 1,224 | 2,025 | 2,784 | 4,474 | 379 | 0.0032 |

Q1, Q2, Q3 represent 25th, 50th, and 75th percentiles. P90, P95, P99 represent 90th, 95th, and 99th percentiles. All reads were basecalled using Dorado (v0.5.2) with trim option. VCaP RNA002 and RNA004 represent matched chemistry comparison.

**Supplementary Table 3** Ablation Study Results for Quality Block

| Model Configuration | F1 Score |
|---|---|
| With Quality Block | **0.99** |
| Without Quality Block | 0.97 |

**Supplementary Table 4** Internal Adapter Prevalence Across Datasets

| Sample | All Reads | | | Chimeric Reads Only | | |
|---|---|---|---|---|---|---|
| | With Internal Adapters (A) | Total (B) | % (A/B) | With Internal Adapters (C) | Total (D) | % (C/D) |
| A549 | 15,690 | 1,703,697 | 0.92 | 10,553 | 12,803 | 82.43 |
| MCF7 | 20,340 | 3,039,468 | 0.67 | 11,115 | 17,646 | 63.00 |
| HCT116 | 57,122 | 4,697,299 | 1.22 | 37,823 | 46,800 | 80.81 |
| K562 | 29,436 | 3,061,722 | 0.96 | 19,289 | 23,214 | 83.09 |
| HepG2 | 22,530 | 1,797,922 | 1.25 | 14,331 | 16,921 | 84.69 |
| VCaP RNA002 | 148,452 | 9,177,422 | 1.62 | 98,878 | 107,265 | 92.18 |
| VCaP RNA004 | 38,878 | 11,714,520 | 0.33 | 6,891 | 29,144 | 23.65 |

Total reads from Dorado with trim. Internal adapters detected by DeepChopper after Dorado processing. VCaP RNA002 and RNA004 demonstrate that adapter-bridged chimeras persist across chemistries.

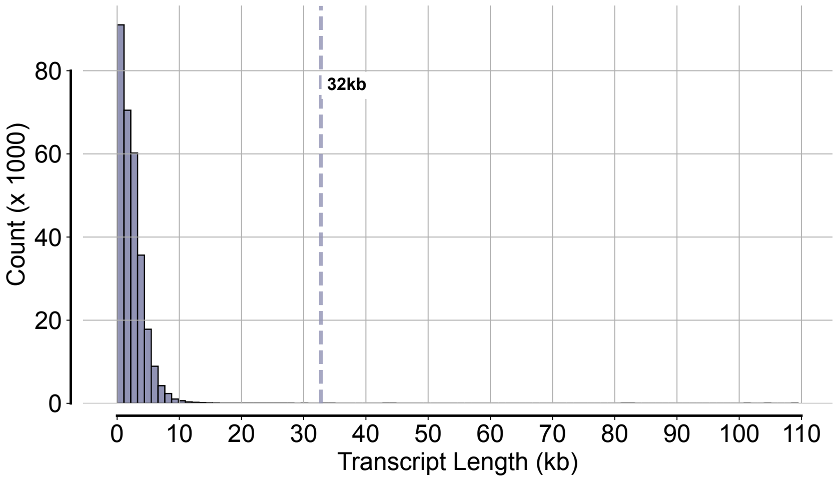


**Supplementary Fig. 1 Distribution of transcript length for protein-coding genes.** Analysis of all protein-coding transcripts from Ensembl GRCh38.115 (released July 2025) shows that >99.99% of transcripts are below the 32 kb threshold (marked with vertical dashed line). The distribution is highly skewed toward shorter transcripts, with median length of ∼2.7 kb.

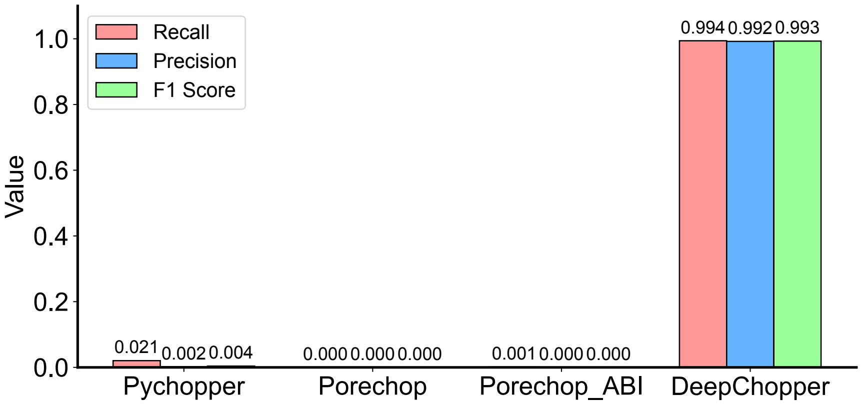


**Supplementary Fig. 2 Performance evaluation in a held-out test dataset** ($N = 60,000$) **showing Recall, Precision, and F1 values for DeepChopper, Pychopper, Porechop, and Porechop_ABI.**

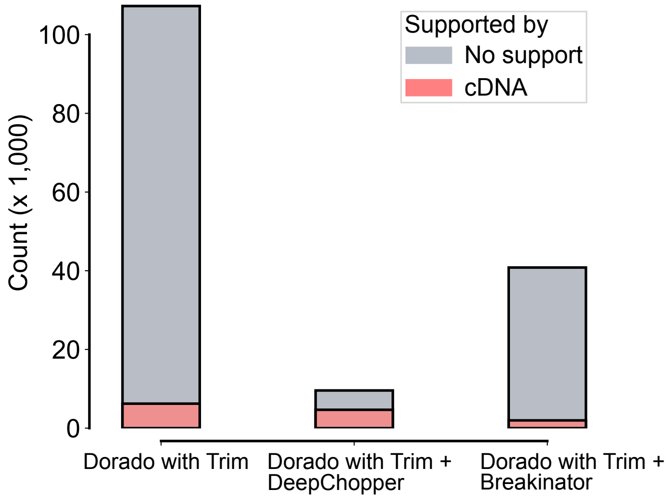


| | Dorado with Trim | Dorado with Trim + DeepChopper | Dorado with Trim + Breakinator |
|---|---|---|---|
| No support | 101,038 | 4,912 | 38,844 |
| cDNA-supported | 6,228 | 4,666 | 1,972 |
| Support Rate | 5.8% | **48.7%** | 4.8% |

**Supplementary Fig. 3 Comparison of chimeric alignment reduction strategies in VCaP RNA002 dRNA-seq data.** Stacked bar plot showing chimeric alignments (in thousands) for three processing pipelines: Dorado with adapter trimming (baseline), Dorado with adapter trimming followed by DeepChopper, and Dorado with adapter trimming followed by Breakinator. Gray bars represent unsupported chimeric alignments (likely artifacts); pink bars represent cDNA-supported chimeric alignments (biological events).

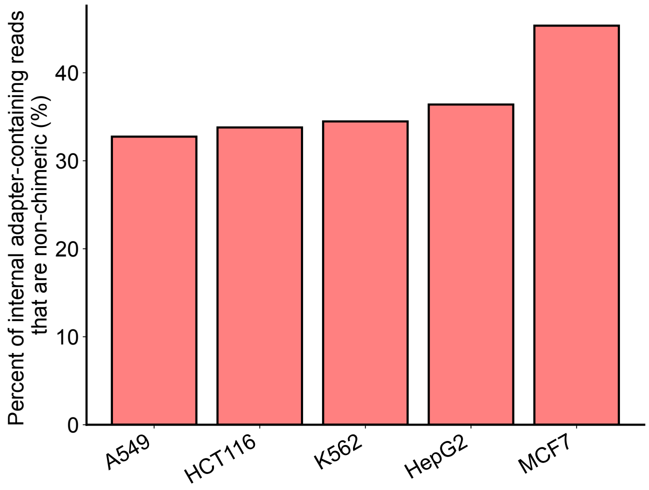


**Supplementary Fig. 4 Percent of internal adapter-containing reads that are non-chimeric** Percentage of internal adapter-containing reads that do not produce chimeric alignments across five human cell lines (RNA002) processed by Dorado with trim followed by DeepChopper. Between 33–45% of adapter-containing reads map as single alignments or fail to map, making them invisible to chimeric alignment-based artifact detection.

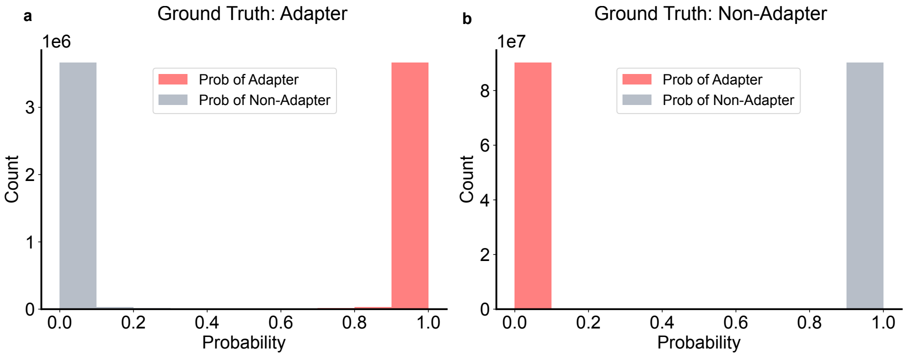


**Supplementary Fig. 5 Prediction probability distributions of DeepChopper for the held-out test dataset ($N = 60,000$).** (a) Distribution of prediction probabilities for sequences with ground truth adapter classification. Red bars represent the probability of adapter prediction, while gray bars show the probability of non-adapter prediction. The count (y-axis) is shown in millions of sequences ($10^6$ scale). (b) Distribution of prediction probabilities for sequences with ground truth non-adapter classification. Red bars indicate the probability of adapter prediction, while gray bars show the probability of non-adapter prediction. The count (y-axis) is shown in tens of millions of sequences ($10^7$ scale). Both distributions demonstrate strong polarization toward correct classification probabilities, indicating the model's high confidence in distinguishing between adapter and non-adapter sequences.

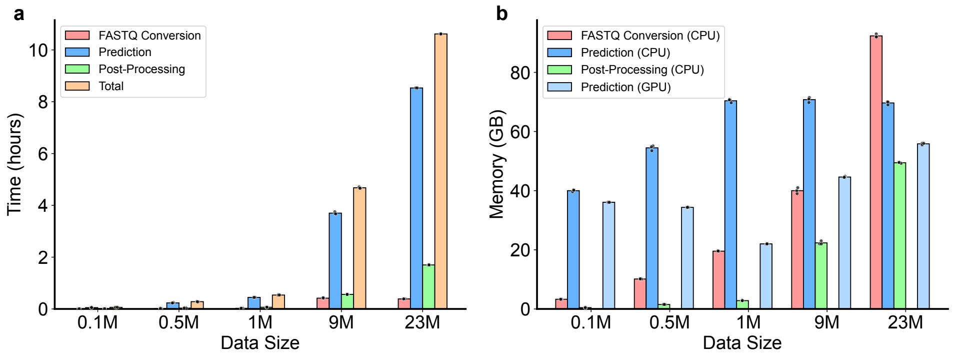


**Supplementary Fig. 6 Computational performance metrics across different data sizes.** All measurements were conducted with three technical replicates. Individual data points are shown as overlaid dots, and bars represent mean values. (a) Runtime analysis showing processing time requirements for different pipeline stages (FASTQ Conversion, Prediction, Post-Processing) and total runtime across five data sizes: subsampled VCaP datasets (0.1M, 0.5M, 1M reads), full VCaP dRNA-seq dataset (9M reads), and merged large-scale dataset (23M reads combining A549, HCT116, HepG2, K562, and MCF7). Runtime scales near-linearly with data size. As data size increases, prediction time becomes the dominant component, requiring approximately 5 hours for the 9M dataset and 10.6 hours for the 23M dataset. (b) Memory usage comparison between CPU and GPU implementations across the same data sizes. The prediction stage shows consistently higher memory requirements. CPU memory usage ranges from 70-93 GB and GPU memory from 34-56 GB across larger datasets, with stable memory footprint indicating no fundamental barriers to processing larger datasets.

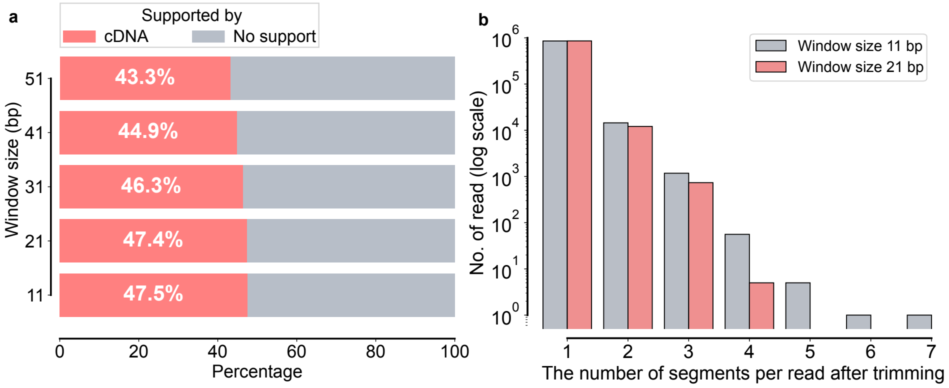


**Supplementary Fig. 7 Effect of window size on chimeric alignment detection and read fragmentation.** (a) Analysis of different sliding window sizes (11, 21, 31, 41, and 51 nucleotides) showing the percentage of cDNA-supported chimeric alignments (red bars) in VCaP. Higher percentages indicate better support. (b) Distribution of the number of segments per read after trimming (x-axis) for window sizes 11 (gray) and 21 (pink), shown on a logarithmic scale (y-axis). Data represents subsampling of 1M reads from the VCaP dataset. Window size 21 maintains similar detection sensitivity to window size 11 while producing fewer fragmented reads.

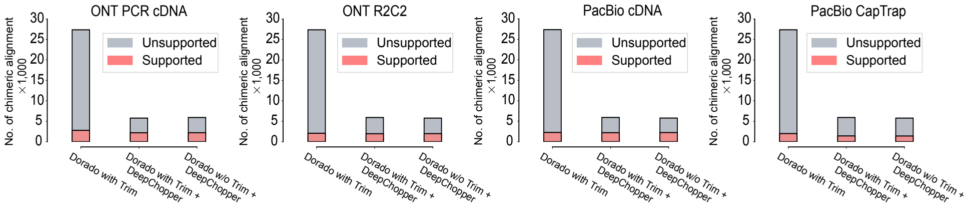


**Supplementary Fig. 8 Chimeric alignments from dRNA-seq of the F121-9 cell line (mouse), evaluated for support using additional Oxford Nanopore Technologies (ONT) and Pacific Biosciences (PacBio) sequencing data with different protocols. DeepChopper-involved methods reduce unsupported chimeric alignments across all methods compared to Dorado with adapter trimming. The bar colors indicate chimeric alignments supported by additional sequencing data (red) and those lacking support (grey).**

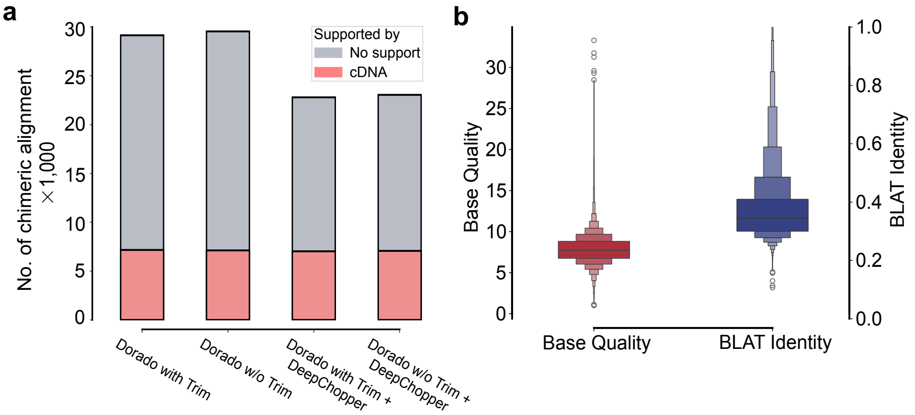


**Supplementary Fig. 9 Evaluation of DeepChopper's predictions on chimeric read artifacts in dRNA-seq data generated using the SQK-RNA004 kit from the VCaP cell line.** (a) Number of chimeric alignments (in thousands) identified in VCaP RNA004 dRNA-seq reads processed by Dorado with and without adapter trimming, Dorado with adapter trimming followed by DeepChopper, and DeepChopper. The bar colors indicate chimeric alignments supported by cDNA sequencing (red) and those lacking support (grey). (b) Base quality scores (left) and BLAST-like alignment tool (BLAT) alignment identity (right) for internal adapter sequences identified by DeepChopper in RNA004 dRNA-seq reads. Enhanced box plots show the median (center line), interquartile range (innermost box, 25th–75th percentiles), and progressively more extreme percentiles (outer boxes). Left: Adapter sequences exhibit low base quality (median = 7.73, IQR = 6.74–8.82, mean = 7.85; $n$ = 11,143). Right: Adapter sequences show poor identity (median = 0.34, IQR = 0.30–0.41, mean = 0.38; $n$ = 6,185), confirming their synthetic, non-biological origin.

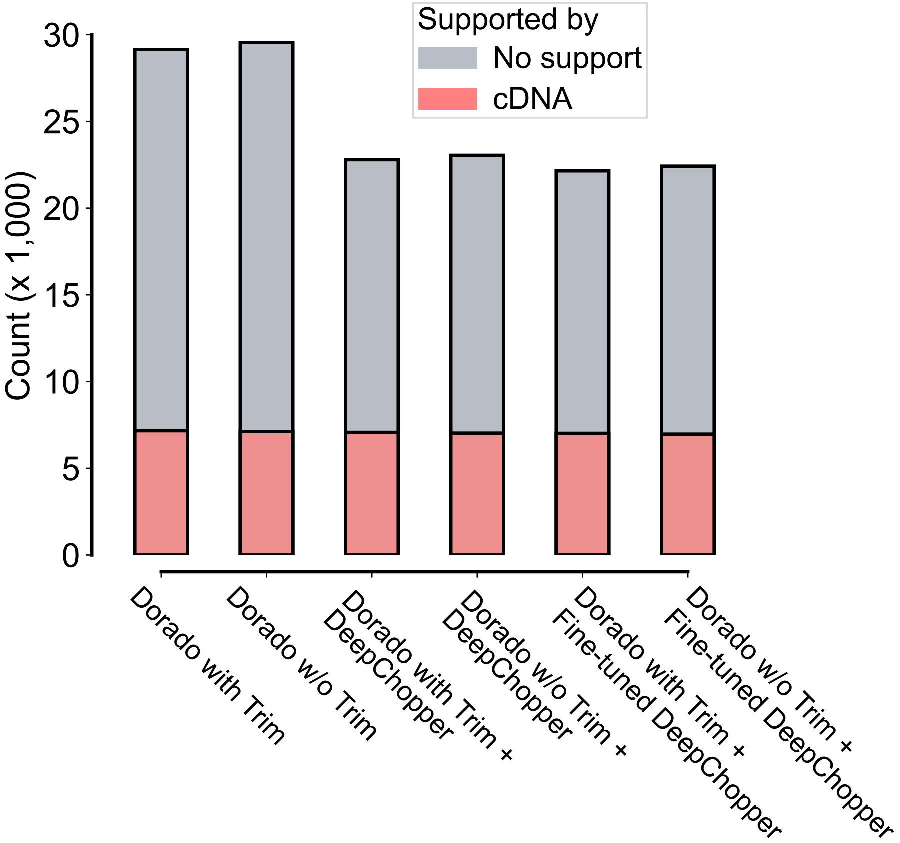


**Supplementary Fig. 10 Performance comparison of original and fine-tuned DeepChopper on RNA004 data.** Number of chimeric alignments (in thousands) identified in VCaP RNA004 dRNA-seq processed under six conditions: Dorado basecalling with and without adapter trimming, followed by original DeepChopper, and followed by fine-tuned DeepChopper. The bar colors indicate chimeric alignments supported by cDNA sequencing (red) and those lacking support (grey).

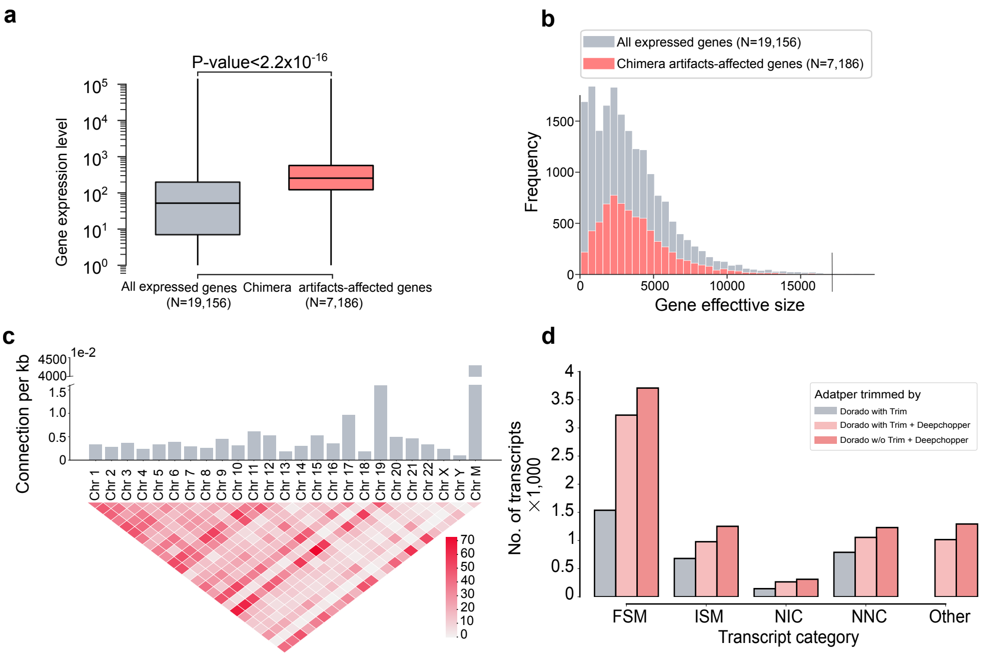


**Supplementary Fig. 11 Analysis of dRNA-seq chimera artifacts and their genomic and transcriptomic characteristics in VCaP cells.** (a) Box plot comparing gene expression levels between all expressed genes (N=19,156) and genes affected by chimera artifacts (N=7,186) in the VCaP dRNA-seq dataset. Chimera artifacts-affected genes exhibit higher expression levels (p-value $< 2.2 \times 10^{-16}$). (b) Distribution of gene effective sizes for all expressed genes and genes affected by chimera artifacts, indicating that the size distributions of genes impacted by chimera artifacts are comparable to those of all expressed genes. (c) Chromosomal distribution and interchromosomal connections from chimeric read artifacts arising from VCaP RNA004 dRNA-seq. The top bar plot shows the number of connections per kilobase for each chromosome, with higher bars indicating more frequent connections. The bottom heatmap visualizes the number of chimeric connections between chromosome pairs, with color intensity representing the connection frequency. (d) Number of detected transcripts across different isoform categories (Full splice match (FSM), Incomplete splice match (ISM), Novel in catalog (NIC), Novel not in catalog (NNC), and Other) from DeepChopper-identified chimeric read artifacts in VCaP RNA004 dRNA-seq data. DeepChopper-corrected reads resulted in a greater number of transcripts compared to adapter-trimmed reads by Dorado across all categories.

**a**

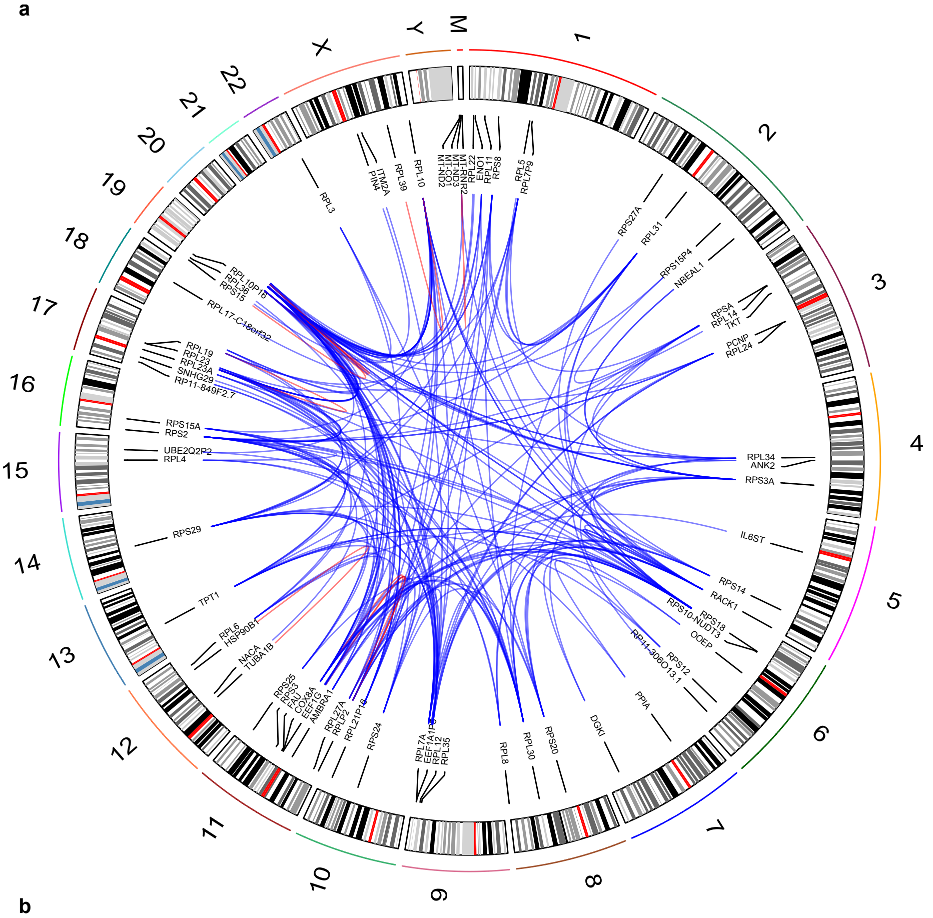


**b**

| Sample | GO Term | Genes | P-value |
|---|---|---|---|
| A549 | Cytoplasmic translation | RPL34, RPS21 | $1.467 \times 10^{-2}$ |
| | Translation | RPL34, RPS21 | $3.040 \times 10^{-2}$ |
| HepG2 | Translation | EEF1A1, RPS19, RPL13, RPS12 | $2.172 \times 10^{-4}$ |
| | Intracellular iron ion homeostasis | MT-RNR2, FTH1, FTL | $7.332 \times 10^{-4}$ |
| | Cytoplasmic translation | RPS19, RPL13, RPS12 | $1.528 \times 10^{-3}$ |
| | Intracellular sequestering of iron ion | FTH1, FTL | $4.250 \times 10^{-3}$ |
| | Translational elongation | EEF1A1, EEF1B2 | $1.150 \times 10^{-2}$ |
| | Iron ion transport | FTH1, FTL | $1.630 \times 10^{-2}$ |
| | Ribosomal small subunit biogenesis | RPS19, RPS12 | $4.526 \times 10^{-2}$ |
| HCT116 | Regulation of translation | RPS3, RPL38, GAPDH | $6.151 \times 10^{-3}$ |
| | Negative regulation of translation | RPS3, RPL13A, GAPDH | $6.525 \times 10^{-3}$ |
| | Cytoplasmic translation | RPL4, RPS6, RPL13A, RPSA, RPL7A, RPS29, RPS3, RPL14, RPLP2, RPL13, RPS20, RPL38, RPS2, RPL28 | $1.008 \times 10^{-24}$ |
| | Translation | RPL4, RPS6, RPL13A, RPSA, EEF1A1, RPL7A, RPS29, RPS3, RPL14, RPLP2, RPL13, RPS20, RPL38, RPS2, RPL28 | $1.566 \times 10^{-22}$ |
| VCaP (SQK-RNA004 kit) | Cytoplasmic translation | RPS16, RPLP2, RPL29 | $7.15 \times 10^{-5}$ |
| | Translation | RPS16, EEF1A1P5, RPLP2, RPL29 | $1.06 \times 10^{-6}$ |

**Supplementary Fig. 12 Analysis of gene fusions derived from chimeric read artifacts in dRNA-seq.** (a) Circos plot depicting chromosomal connections of gene fusions resulting from chimeric read artifacts in VCaP cells. Blue lines represent inter-chromosomal fusion events, while red lines indicate intra-chromosomal fusions. The outer track displays chromosomal ideograms labeled with respective chromosome numbers. (b) Gene Ontology (GO) enrichment analysis of fusion genes derived from chimeric read artifacts identified by DeepChopper in dRNA-seq data from A549, HepG2, and HCT116 cell lines, and VCaP RNA004 dRNA-seq data. The table lists enriched GO terms of biological processes, associated genes, and the statistical significance (p-values) for each enrichment.

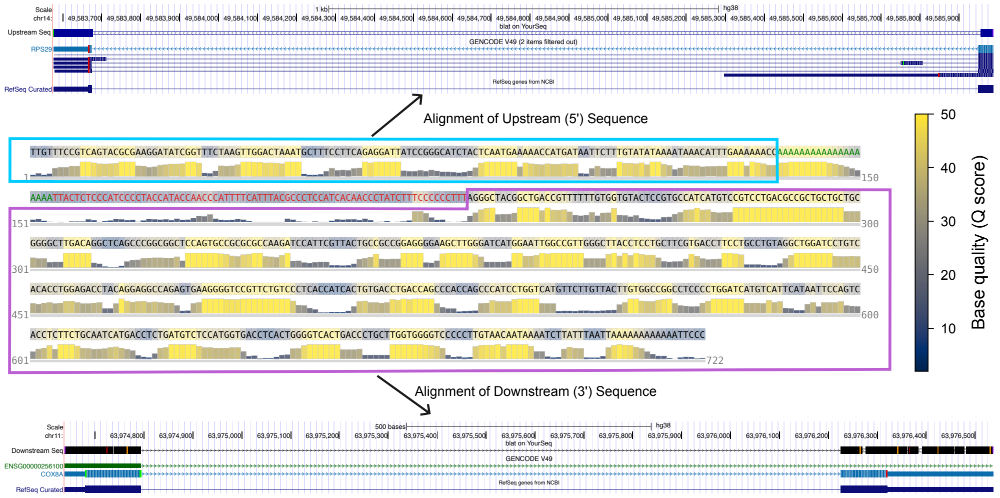


**Supplementary Fig. 13 Representative internal adapter detection with base quality visualization.** Representative read (Fig. 3e) from VCaP RNA002 processed with Dorado trim followed by DeepChopper, demonstrating internal adapter detection (highlighted in red, positions 155-229) and sub-reads recovery (blue box and purple box). (upper panel) BLAT alignment of upstream sequence (blue box, positions 1–154) to chr14 (RPS29 gene) with 0.98 identity, confirming genuine biological RNA. (middle panel) Full read visualization showing internal adapter and sub-reads. Base quality scores ($Q$ scores) shown as color-coded bars: yellow indicates high quality ($Q > 40$), dark blue indicates low quality ($Q < 10$). The adapter region shows characteristic poly-A sequences upstream (highlighted in green) and lower base quality compared to flanking biological sequences. The adapter region (75 bp) shows no matches found to the reference genome. Sequences before and after the adapter represent genuine biological RNA from different transcripts artificially joined during library preparation or basecalling. Read ID: 3b2292e9-43e5-4e40-87d9-ccc23897377c. (bottom panel) BLAT alignment of downstream sequence (purple box, positions 230–722) to chr11 (COX8A gene) with 0.96 identity, confirming genuine biological RNA from a different transcript.

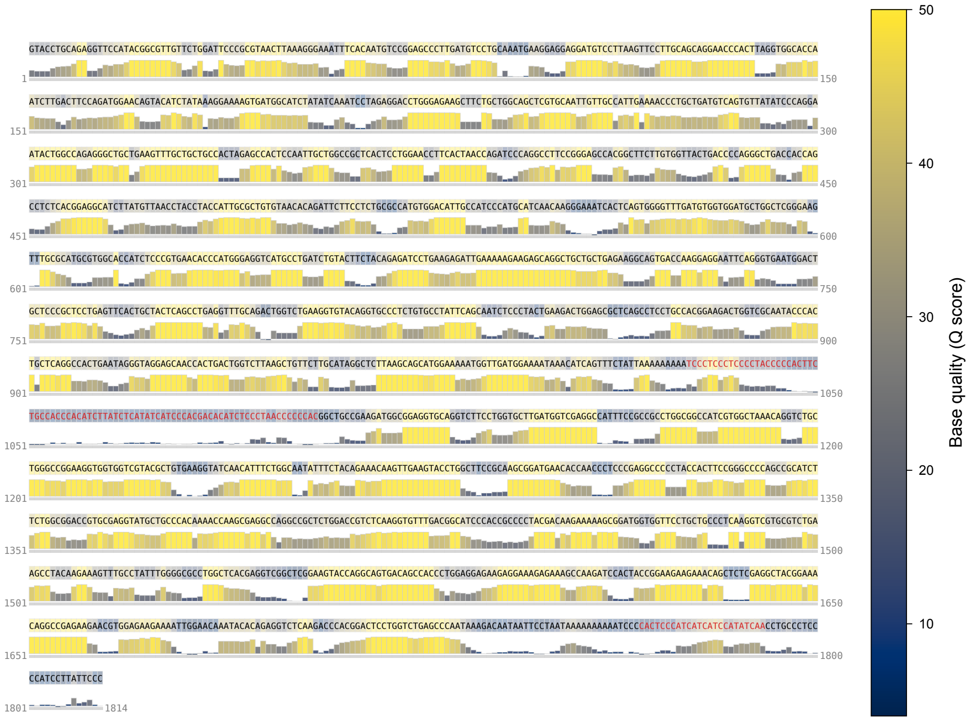

**Supplementary Fig. 14 Challenge Scenario 1: Incomplete 3' terminal adapter detection in multi-adapter read.** Representative read from VCaP RNA002 processed with Dorado without trim followed by DeepChopper (with refinement applied). The read contains both an internal adapter (positions 1026-1105, highlighted in red, correctly detected) and a 3' end adapter that DeepChopper failed to completely detect. Base quality scores ($Q$ scores) shown as color-coded bars: yellow indicates high quality ($Q > 40$), dark blue indicates low quality ($Q < 10$). The internal adapter shows characteristic low quality compared to flanking biological sequences. This scenario demonstrates that when multiple adapters are present, DeepChopper reliably detects internal adapters (its primary function) but may incompletely detect terminal adapters. Read ID: c16c6ade-135b-4073-a1d6-5a9c6900bfb2.

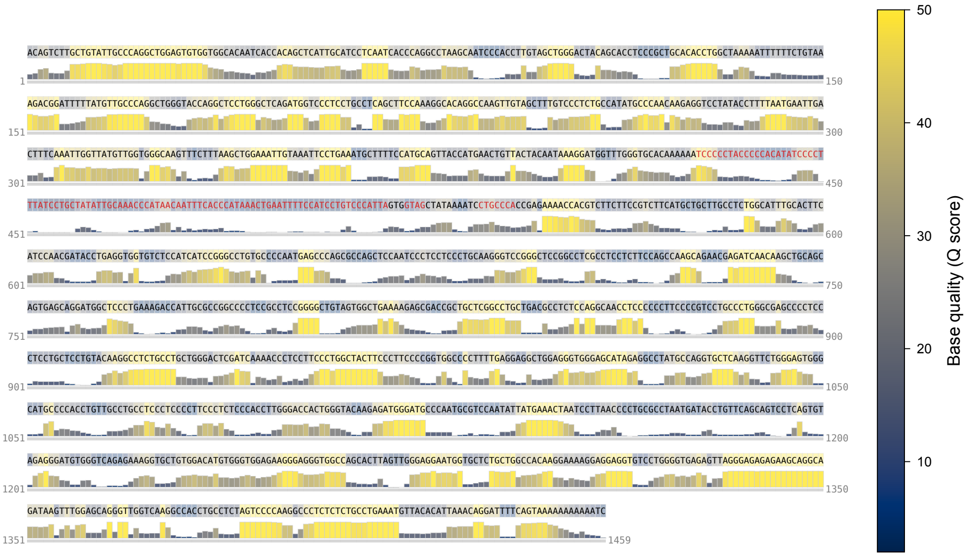

**Supplementary Fig. 15 Challenge Scenario 2: Partial internal adapter detection.** Representative read from VCaP RNA002 processed with Dorado trim option followed by DeepChopper (with refinement applied). The read shows partial adapter detection (highlighted in red), demonstrating challenges with degraded or error-rich adapter sequences where DeepChopper may detect only portions of the complete adapter sequence. The sequences GTG (positions 519-521) and CTATAAAATC (positions 526-535) may be part of internal adapter. Base quality scores ($Q$ scores) shown as color-coded bars: yellow indicates high quality ($Q > 40$), dark blue indicates low quality ($Q < 10$). Such partial detections can lead to spurious short fragments, which are addressed through post-processing length filtering. Read ID: 0955d980-9c79-48c2-a474-08c8c39cb00f.

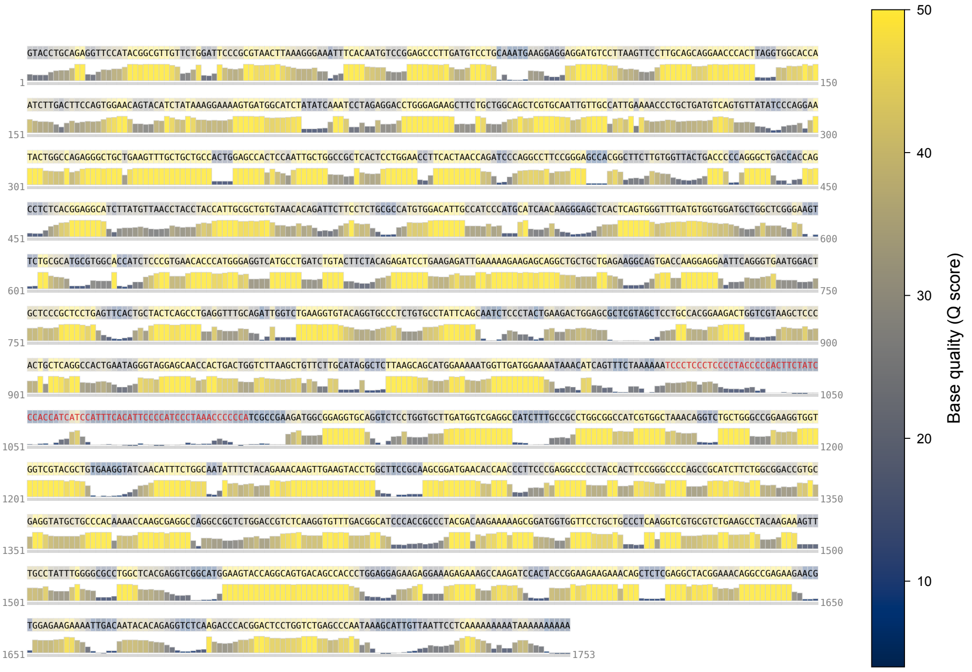


**Supplementary Fig. 16 Solution for Challenge Scenario 1: Combined Dorado-DeepChopper workflow.** The same representative read (ID: c16c6ade-135b-4073-a1d6-5a9c6900bfb2) processed with Dorado with trim followed by DeepChopper. Dorado successfully removed the 3' end adapter, while DeepChopper detected the internal adapter (highlighted in red, positions adjusted after Dorado trimming). Base quality scores ($Q$ scores) shown as color-coded bars: yellow indicates high quality ($Q > 40$), dark blue indicates low quality ($Q < 10$). The internal adapter shows characteristic low quality. This demonstrates that combining Dorado (3' end adapters) with DeepChopper (internal adapters) addresses complementary problems and resolves the incomplete detection issue shown in Challenge Scenario 1. (Supplementary Fig. 14)
