# Supplementary material for: Genomic language model mitigates chimera artifacts in nanopore direct RNA sequencing
Source: Nat Commun. 2026 Jan 19;17:1864. doi: 10.1038/s41467-026-68571-5 (PMC12923543; doi:10.1038/s41467-026-68571-5)
Supplement: Supplementary file 2 — Reporting Summary [file 41467_2026_68571_MOESM2_ESM.pdf]

nature portfolio

Corresponding author(s): Rendong Yang

Last updated by author(s): Dec 28, 2025

Reporting Summary

Nature Portfolio wishes to improve the reproducibility of the work that we publish. This form provides structure for consistency and transparency in reporting. For further information on Nature Portfolio policies, see our Editorial Policies and the Editorial Policy Checklist.

Statistics

For all statistical analyses, confirm that the following items are present in the figure legend, table legend, main text, or Methods section.

n/a	Confirmed	
☐	☒	The exact sample size (*n*) for each experimental group/condition, given as a discrete number and unit of measurement
☐	☒	A statement on whether measurements were taken from distinct samples or whether the same sample was measured repeatedly
☐	☒	The statistical test(s) used AND whether they are one- or two-sided<br>*Only common tests should be described solely by name; describe more complex techniques in the Methods section.*
☒	☐	A description of all covariates tested
☒	☐	A description of any assumptions or corrections, such as tests of normality and adjustment for multiple comparisons
☐	☒	A full description of the statistical parameters including central tendency (e.g. means) or other basic estimates (e.g. regression coefficient) AND variation (e.g. standard deviation) or associated estimates of uncertainty (e.g. confidence intervals)
☐	☒	For null hypothesis testing, the test statistic (e.g. *F*, *t*, *r*) with confidence intervals, effect sizes, degrees of freedom and *P* value noted<br>*Give P values as exact values whenever suitable.*
☒	☐	For Bayesian analysis, information on the choice of priors and Markov chain Monte Carlo settings
☒	☐	For hierarchical and complex designs, identification of the appropriate level for tests and full reporting of outcomes
☒	☐	Estimates of effect sizes (e.g. Cohen's *d*, Pearson's *r*), indicating how they were calculated

Our web collection on statistics for biologists contains articles on many of the points above.

Software and code

Policy information about availability of computer code

Data collection	we did not use any software to collect data, and the data is downloaded from from the SG-NEx project (https://github.com/GoekeLab/sg-nex-data), the Cancer Cell Line Encyclopedia (CCLE) project (https://sites.broadinstitute.org/ccle) and the Long-read RNA-Seq Genome Annotation Assessment Project (LRGASP) (https://www.gencodegenes.org/pages/LRGASP)
Data analysis	The program is open<br>source and available on GitHub (https://github.com/ylab-hi/DeepChopper) under the<br>Apache License, Version 2.0. The package can be installed via PyPI (https://pypi.<br>org/project/deepchopper/) using pip, with wheel distributions provided for Windows,<br>Linux, and macOS.<br>An interactive demo<br>is available on Hugging Face (https://huggingface.co/spaces/yangliz5/deepchopper),<br>allowing users to test DeepChopper's functionality without local installation. The direct cDNA data in FAST5 format were converted to POD5 format using the POD5 conversion tool (https://pod5-file-format.readthedocs.io).<br>Subsequently, FASTQ files were generated using Dorado (v0.5.2) with adapter trimming enabled.

For manuscripts utilizing custom algorithms or software that are central to the research but not yet described in published literature, software must be made available to editors and reviewers. We strongly encourage code deposition in a community repository (e.g. GitHub). See the Nature Portfolio guidelines for submitting code & software for further information.

Data

Policy information about availability of data

All manuscripts must include a data availability statement. This statement should provide the following information, where applicable:

- Accession codes, unique identifiers, or web links for publicly available datasets
- A description of any restrictions on data availability
- For clinical datasets or third party data, please ensure that the statement adheres to our policy

All raw and processed data generated in this study have been deposited in the Gene Expression Omnibus (GEO) under accession number GSE277934, including:

- Direct RNA sequencing (dRNA-seq) using SQK-RNA002 and SQK-RNA004 kits
- Direct cDNA sequencing of VCaP cells

A secure token (sdwfckwmbzqdbyx) is available for reviewers to access these data.

We also utilized the following publicly available datasets:

1. Direct cDNA sequencing data for six cancer cell lines (A549, K562, HepG2, MCF7, and HCT116) from the SG-NEx project (https://github.com/GoekeLab/sg-nex-data)

2. Short-read RNA-seq data for the VCaP cell line from the Cancer Cell Line Encyclopedia (CCLE) project (https://sites.broadinstitute.org/ccle), available under SRA accession SRX5417211

3. Long-read sequencing data from the WTC11 and F121-9 cell lines, obtained from the Long-read RNA-Seq Genome Annotation Assessment Project (LRGASP) (https://www.gencodegenes.org/pages/LRGASP/), including:
   - ONT PCR-cDNA
   - ONT CapTrap
   - ONT R2C2 (FASTA format)
   - PacBio cDNA
   - PacBio CapTrap

Research involving human participants, their data, or biological material

Policy information about studies with human participants or human data. See also policy information about sex, gender (identity/presentation), and sexual orientation and race, ethnicity and racism.

Reporting on sex and gender	No human research participants are involved in this work
Reporting on race, ethnicity, or other socially relevant groupings	No human research participants are involved in this work
Population characteristics	No human research participants are involved in this work
Recruitment	No human research participants are involved in this work
Ethics oversight	No human research participants are involved in this work

Note that full information on the approval of the study protocol must also be provided in the manuscript.

Field-specific reporting

Please select the one below that is the best fit for your research. If you are not sure, read the appropriate sections before making your selection.

☒ Life sciences ☐ Behavioural & social sciences ☐ Ecological, evolutionary & environmental sciences

For a reference copy of the document with all sections, see nature.com/documents/nr-reporting-summary-flat.pdf

Life sciences study design

All studies must disclose on these points even when the disclosure is negative.

Sample size	The data were derived from the SG-NEx project, which comprises six human cell lines: HEK293T (embryonic kidney), A549 (lung carcinoma), K562 (leukemia), HepG2 (liver cancer), MCF7 (breast cancer), and HCT116 (colorectal cancer). All six cell lines were incorporated into the training set, with detailed methodological procedures described in the Methods section.
Data exclusions	No exclusions were made.

Replication	Not applicable since the study does not involved wet lab experiments.
Randomization	No randomization was performed. However, this was not relevant to our study because it does not involve case/control comparisons and statistical testing of hypotheses.
Blinding	The study design does not involved blinding. However, this was not relevant to our study because it does not involve case/control comparisons and statistical testing of hypotheses.

Reporting for specific materials, systems and methods

We require information from authors about some types of materials, experimental systems and methods used in many studies. Here, indicate whether each material, system or method listed is relevant to your study. If you are not sure if a list item applies to your research, read the appropriate section before selecting a response.

Materials & experimental systems

n/a	Involved in the study
☒	☐ Antibodies
☐	☒ Eukaryotic cell lines
☒	☐ Palaeontology and archaeology
☒	☐ Animals and other organisms
☒	☐ Clinical data
☒	☐ Dual use research of concern
☒	☐ Plants

Methods

n/a	Involved in the study
☒	☐ ChIP-seq
☒	☐ Flow cytometry
☒	☐ MRI-based neuroimaging

Eukaryotic cell lines

Policy information about cell lines and Sex and Gender in Research

Cell line source(s)	VCaP cell line (Male) was obtained from American Type Culture Collection (ATCC, Cat# CRL-2876).
Authentication	Short tandem repeat profiling was used to verify the identity of VCaP cell line at the beginning of the study.
Mycoplasma contamination	The VCaP cell line was tested for mycoplasma contamination quarterly using PCR-based detection method. All tests were negative.
Commonly misidentified lines (See ICLAC register)	VCaP cell line used in this study is not listed in the ICLAC database of commonly misidentified cell lines.

Plants

Seed stocks	No plant is involved in this work.
Novel plant genotypes	No plant is involved in this work.
Authentication	No plant is involved in this work.
